# Socioeconomic Inequalities in Oral Frailty and Its Components

**DOI:** 10.1111/odi.70169

**Published:** 2025-12-16

**Authors:** Duc Sy Minh Ho, Yusuke Matsuyama, Sakura Kiuchi, Tatsuo Yamamoto, Jun Aida

**Affiliations:** ^1^ Department of Dental Public Health, Graduate School of Medical and Dental Sciences Institute of Science Tokyo Tokyo Japan; ^2^ Faculty of Odonto‐Stomatology, Hue University of Medicine and Pharmacy, Hue University Hue Vietnam; ^3^ Department of International and Community Oral Health, Graduate School of Dentistry Tohoku University Miyagi Japan; ^4^ Frontier Research Institute for Interdisciplinary Sciences Tohoku University Miyagi Japan; ^5^ Department of Preventive Dentistry and Dental Public Health Kanagawa Dental University Yokosuka Japan

**Keywords:** health inequalities, oral frailty, socioeconomic factors

## Abstract

**Objectives:**

Oral frailty, an intermediate stage between healthy and declined oral function, involves impairments such as tooth loss and chewing difficulty. Socioeconomic conditions may influence oral frailty risk. This study examined the socioeconomic inequalities in oral frailty and its components among older adults in Japan.

**Methods:**

This cross‐sectional study used 2022 questionnaire data from the Japan Gerontological Evaluation Study of adults aged ≥ 65 years. Dependent variables included oral frailty and its five components. Oral frailty was defined as having ≥ 2 of the following: fewer teeth, dry mouth, and difficulty in chewing, swallowing, or speaking. Independent variables were socioeconomic factors, comprising educational attainment, income, wealth, and pension type. Age and sex were adjusted as confounders. The Slope Index of Inequality (SII) and Relative Index of Inequality (RII) were estimated after applying multiple imputation to address missing data.

**Results:**

A total of 21,924 older adults were included. The prevalence of oral frailty was 33.6%. Significant inequalities were identified across all socioeconomic factors. Wealth showed the greatest inequality, with an adjusted SII of 0.17 (95% confidence interval (CI): 0.14–0.20) and RII of 1.66 (95% CI: 1.52–1.82).

**Conclusions:**

Substantial socioeconomic inequalities in oral frailty were observed, with wealth being the most influential factor.

## 1 Introduction

Oral frailty has emerged as a critical public health concern, particularly among older adults (Dibello et al. 2021; Tanaka et al. 2024). In 2024, a consensus statement from three leading Japanese academic societies defined oral frailty, characterised by a decline in functions such as chewing, swallowing, and speaking, as an intermediate state between normal oral health and functional deterioration (Tanaka et al. 2024). Research interest in oral frailty has expanded considerably, encompassing its prevalence and assessment tools (de Sire et al. 2022). Two large studies employing the Oral Frailty 5‐Item Checklist (OF‐5), a validated tool, have reported the prevalence of 36.7% and 39.3% among community‐dwelling older adults, respectively (Iwasaki et al. 2024; Tanaka et al. 2024). Moreover, oral frailty has been linked to adverse outcomes such as cognitive decline and mortality, leading to a diminished quality of life (Dibello et al. 2021; Yang et al. 2024).

Oral health inequalities, which present as a social gradient in oral health corresponding to socioeconomic status (SES), are well‐documented (Peres et al. 2019). A systematic review of 155 studies found that those with lower education levels had more dental caries (Schwendicke et al. 2015). Meta‐analyses have shown that a lower income is associated with an increased risk of dental caries and tooth loss (Singh et al. 2019).

While discussions on socioeconomic inequalities typically center around the most prevalent oral diseases worldwide—such as tooth decay and tooth loss (Peres et al. 2019; Watt 2023)—the patterns of socioeconomic inequalities within the emerging issue of oral frailty remain underexplored. A recent study in Japan reported regional disparities in oral frailty among older adults, with rural areas exhibiting higher prevalence than metropolitan regions (Yamamoto et al. 2024). In addition, another Japanese study revealed inequalities of oral frailty by income and education (Fujita et al. 2025). These findings suggest that oral frailty may also be shaped by socioeconomic determinants. Therefore, this study aims to investigate socioeconomic inequalities in oral frailty and its individual components among older adults in Japan, and further examine how disparities in these components contribute to the overall inequality in oral frailty.

## 2 Methods

### 2.1 Data Collection

The cross‐sectional study employed data from the 2022 wave of the Japan Gerontological Evaluation Study (JAGES) (Kondo 2016; Kondo and Rosenberg 2018), which investigates social determinants of health, health inequalities, and preventive strategies among independent adults aged 65 and over.

Our analysis focused on a final sample of 21,924 older adults from 70 municipalities, drawn from the 2022 JAGES survey. These participants completed Version D of the questionnaire, which specifically assessed oral health behaviour, including questions on oral frailty components. The full survey included a core questionnaire capturing demographic and general health information, along with eight supplementary versions (A to H), each covering different health domains. To ensure representativeness, these version‐specific questionnaires were randomly distributed to approximately one‐eighth of the total participants. Although our dataset represents a subset of the 2022 wave of the JAGES study, the sample size is sufficiently large and geographically diverse to support robust statistical analyses across socioeconomic subgroups. The random assignment of questionnaire versions and inclusion of 70 municipalities enhance the generalisability of our findings.

In total, the 2022 JAGES survey was administered to 309,833 older adults across 72 municipalities. It achieved a response rate of 66.6%, with 206,204 responses received. After excluding 13,159 responses due to invalid data or lack of consent, 193,045 valid responses remained. From these, we selected respondents who completed Version D (*n* = 24,092), then excluded individuals requiring assistance with daily living or with missing data on this variable, resulting in the final analytical sample of 21,924. The participant selection process is illustrated in Figure S1.

### 2.2 Dependent Variables

The dependent variables in this study were oral frailty and its components. In previous studies conducted in Japan, oral frailty was commonly assessed using the OF‐5—a simple checklist developed to allow older adults to perform self‐checks and to enable non‐dental healthcare professionals to assess the oral conditions of patients or individuals receiving care (Iwasaki et al. 2024; Tanaka et al. 2024). The OF‐5 includes five components: fewer teeth, difficulty in chewing, difficulty in swallowing, dry mouth, and low articulatory oral motor skills. The original fifth item in the OF‐5—low articulatory oral motor skills, assessed by the question ‘Have you had difficulty with clear pronunciation recently?’—was not available in the 2022 JAGES survey. To maintain consistency with the OF‐5 framework, we substituted this item with a comparable question available in the dataset: ‘In the past six months, have you had difficulty speaking?’, with a ‘yes’ response indicating difficulty in speaking. As a result, a modified version of the OF‐5 was employed in this study. The five components were defined as follows: fewer teeth was defined as having 0–19 natural teeth; difficulty in chewing as a ‘yes’ response to having more difficulty eating tough foods compared to 6 months ago; difficulty in swallowing as a ‘yes’ response to choking on tea or soup recently; dry mouth as a ‘yes’ response to frequently experiencing dry mouth; and difficulty in speaking as a ‘yes’ response to having speaking difficulties in the past 6 months.

Oral frailty was defined as the presence of two or more of the five components and treated as a binary variable, consistent with the OF‐5 definition (Tanaka et al. 2024). Binary indicators representing the conditions for each component were also included.

### 2.3 Independent Variables

The independent variables were socioeconomic factors, including educational attainment, equivalent annual household income, wealth, and pension type, based on prior studies (Morohoshi et al. 2024; Sasaki et al. 2018). Educational attainment was classified as ≤ 9 years, 10–12 years, or ≥ 13 years. Income was grouped into five categories: < 1 million yen, 1–1.99 million, 2–2.99 million, 3–3.99 million, and ≥ 4 million yen. Wealth was categorized as < 1 million yen, 1–4.99 million, 5–9.99 million, 10–49.9 million, and ≥ 50 million yen. Pension type was classified into four groups. The None group included individuals without pension benefits. The Poor group consisted of part‐time workers, self‐employed individuals, and unemployed persons covered only by the National Pension Plan. The Moderate group comprised government employees and full‐time workers at small‐ to medium‐sized enterprises enrolled in the Employees' or Mutual‐Aid Pension Plans, with or without National Pension coverage. The Rich group primarily included full‐time employees of large corporations receiving Corporate or Personal Pension Plans, in addition to possible coverage under one or more of the aforementioned pension schemes.

### 2.4 Confounding Variables

Age and sex were included as confounders in this study. Age was categorised into five groups: 65–69, 70–74, 75–79, 80–84, and ≥ 85 years. Sex was classified as male or female.

### 2.5 Statistical Analysis

Figure S2 presents the hypothesised causal framework examined in this study. First, descriptive analyses were conducted to investigate the characteristics of the participants based on the prevalence of oral frailty. In addition, the Slope Index of Inequality (SII) and the Relative Index of Inequality (RII) were employed to identify the socioeconomic inequalities in oral frailty and its components. These measures have been widely applied to evaluate health disparities across various domains in older adults (Morohoshi et al. 2024; Shang and Wei 2023). The SII reflects the estimated difference in the prevalence of oral frailty between the highest and lowest socioeconomic groups, while the RII calculates the ratio of these two values. In our analysis, the SII and RII were estimated using the Ridit score (Ernstsen et al. 2012), which was obtained from the ‘riigen’ command in Stata (Kroll 2013). The SII and RII for oral frailty and its components were calculated using two models. The first model adjusted for age and sex, with each socioeconomic factor included independently. The second model simultaneously included all socioeconomic factors, adjusting for the same confounders.

The contribution of each oral frailty component to socioeconomic inequalities in oral frailty was estimated by calculating the percentage reduction in the SII coefficient across different adjusted models (Singh‐Manoux et al. 2005).

$$\begin{array}{c} \text{Percentage reduction in}\;\mathrm{SII}\\ =\frac{\left(\text{SIIsocioeconomic factor},\text{controlling for}\;\mathrm{age}\;\text{and}\;\mathrm{sex}-\text{SIIsocioeconomic factor},\text{controlling for}\;\mathrm{age},\mathrm{sex},\text{and}\;\mathrm{one}\;\text{oral frailty component}\right)\;\times 100\;}{\text{SIIsocioeconomic factor},\text{controlling for}\;\mathrm{age}\;\text{and}\;\mathrm{sex}} \end{array}$$

Table S1 presents the number and proportion of missing variables. To address missing data and minimise bias, multiple imputation (MI) was conducted under the assumption that data were missing at random (Sterne et al. 2009). Using chained equations, 20 imputed datasets were created. The imputation model included independent and dependent variables as well as confounders (Woods et al. 2024). Final estimates were obtained by pooling the results across imputed datasets using Rubin's rules (Rubin 1996).

In addition, a complete case analysis was performed as a sensitivity analysis, focusing on participants with no missing data. All analyses were conducted using Stata version 18.0 (StataCorp, College Station, TX, USA). A two‐sided *p* < 0.05 was considered statistically significant. This study adhered to the STROBE and OHStat reporting guidelines.

## 3 Results

**FIGURE 1:**
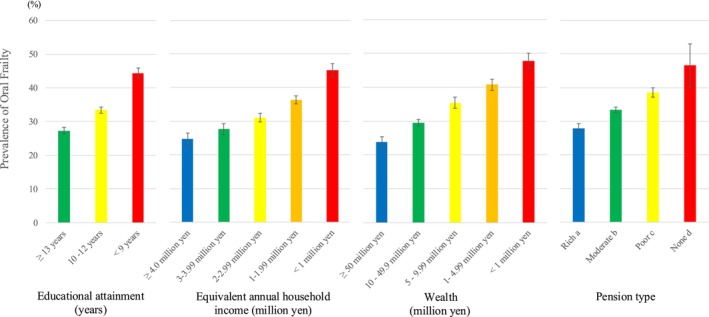
The prevalence of oral frailty by educational attainment, equivalent annual household income, wealth, and pension type (*n* = 21,924). a: Enrolled in Corporate Pension Plan or Personal Pension Plan, either with or without National Pension Plan, Employees' Pension Plan or Mutual‐Aid Pension Plan. b: Enrolled in Employees' Pension Plan or Mutual‐Aid Pension Plan, either with or without National Pension Plan. c: Enrolled in National Pension Plan. d: No pension benefits. Error bars represent the 95% confidence interval.

A total of 21,924 older adults were included in the analysis. The mean age of the participants was 74.8 (SD = 6.3) years, with 52.3% being female. The prevalence of oral frailty was 33.6%. Figure 1 illustrates the oral frailty prevalence across four socioeconomic indicators, all of which showed clear social gradients.

Table 1 presents the demographic characteristics of participants stratified by oral frailty status after MI. Tables S2–S6 display the corresponding characteristics stratified by each component of oral frailty. Overall, the prevalence of oral frailty and its components was higher among individuals with lower education, income, wealth, and less favorable pension types.

**TABLE 1:** Demographic characteristics of participants, stratified by the prevalence of oral frailty after multiple imputation (*n* = 21,924).

Characteristics	Oral frailty^a^		
	Absence	Presence	Overall
	(row %^b^)	(row %^b^)	No. (col %^c^)
	(*n* = 14,554)	(*n* = 7370)	(*n* = 21,924)
*Age*			
65–69 years	75.3%	24.7%	5088 (23.2%)
70–74 years	68.8%	31.2%	6542 (29.8%)
75–79 years	63.8%	36.2%	5032 (23.0%)
80–84 years	60.0%	40.0%	3495 (15.9%)
≥ 85 years	51.9%	48.1%	1767 (8.1%)
*Sex*			
Male	65.6%	34.4%	10,459 (47.7%)
Female	67.1%	32.9%	11,465 (52.3%)
*Educational attainment*			
≥ 13 years	72.7%	27.3%	7588 (34.6%)
10–12 years	66.6%	33.4%	9667 (44.1%)
≤ 9 years	55.6%	44.4%	4669 (21.3%)
*Equivalent annual household income*			
≥ 4.0 million yen	75.3%	24.7%	2582 (11.8%)
3–3.99 million yen	72.4%	27.6%	3241 (14.8%)
2–2.99 million yen	69.0%	31.0%	5132 (23.4%)
1–1.99 million yen	63.7%	36.3%	7983 (36.4%)
< 1 million yen	54.9%	45.1%	2986 (13.6%)
*Wealth*			
≥ 50 million yen	76.2%	23.8%	3024 (13.8%)
10–49.9 million yen	70.5%	29.5%	9124 (41.6%)
5–9.99 million yen	64.6%	35.4%	3636 (16.6%)
1–4.99 million yen	59.2%	40.8%	3764 (17.2%)
< 1 million yen	52.2%	47.8%	2376 (10.8%)
*Pension type*			
Rich^d^	72.1%	27.9%	4279 (19.5%)
Moderate^e^	66.6%	33.4%	12,642 (57.7%)
Poor^f^	61.4%	38.6%	4758 (21.7%)
None^g^	53.5%	46.5%	245 (1.1%)

^a^ Oral frailty was defined by the presence of two or more of these components: fewer teeth, difficulty in chewing, difficulty in swallowing, dry mouth, and difficulty in speaking.

^b^ Row percentage.

^c^ Column percentage.

^d^ Enrolled in Corporate Pension Plan or Personal Pension Plan, either with or without National Pension Plan, Employees' Pension Plan, or Mutual‐Aid Pension Plan.

^e^ Enrolled in Employees' Pension Plan or Mutual‐Aid Pension Plan, either with or without National Pension Plan.

^f^ Enrolled in National Pension Plan.

^g^ No pension benefits.

Table 2 presents the SII and RII for oral frailty by each socioeconomic factor after MI. In the age‐ and sex‐adjusted model, all socioeconomic indicators showed statistically significant inequalities. These associations remained significant in the fully adjusted model, where all socioeconomic indicators were included simultaneously. Among them, wealth exhibited the largest disparities, with an adjusted SII of 0.17 (95% CI: 0.14–0.20) and an adjusted RII of 1.66 (95% CI: 1.52–1.82). Tables S7–S11 show the SII and RII for each oral frailty component, all of which consistently demonstrated the greatest socioeconomic inequalities in the domain of wealth.

**TABLE 2:** The Slope Index of Inequality (SII) and Relative Index of Inequality (RII) of socioeconomic factors in the prevalence of oral frailty after multiple imputation (*n* = 21,924).

	Age‐ and sex‐adjusted model^a^				Fully‐adjusted model^b^			
	SII^c^		RII^d^		SII		RII	
	95% CI^e^	*p*	95% CI	*p*	95% CI	*p*	95% CI	*p*
Educational attainment	0.18 (0.16; 0.21)	< 0.01	1.69 (1.57; 1.82)	< 0.01	0.11 (0.08; 0.13)	< 0.01	1.34 (1.25; 1.45)	< 0.01
Equivalent annual household income	0.19 (0.16; 0.21)	< 0.01	1.75 (1.62; 1.89)	< 0.01	0.07 (0.04; 0.10)	< 0.01	1.24 (1.13; 1.36)	< 0.01
Wealth	0.25 (0.22; 0.27)	< 0.01	2.04 (1.89; 2.20)	< 0.01	0.17 (0.14; 0.20)	< 0.01	1.66 (1.52; 1.82)	< 0.01
Pension type	0.12 (0.09; 0.14)	< 0.01	1.43 (1.32; 1.54)	< 0.01	0.05 (0.03; 0.08)	< 0.01	1.17 (1.08; 1.26)	< 0.01

^a^ Adjusted for age and sex. Each socioeconomic factor was separately included.

^b^ Adjusted for age, sex, and other socioeconomic factors.

^c^ Slope Index of Inequality.

^d^ Relative Index of Inequality.

^e^ Confidence interval.

**FIGURE 2:**
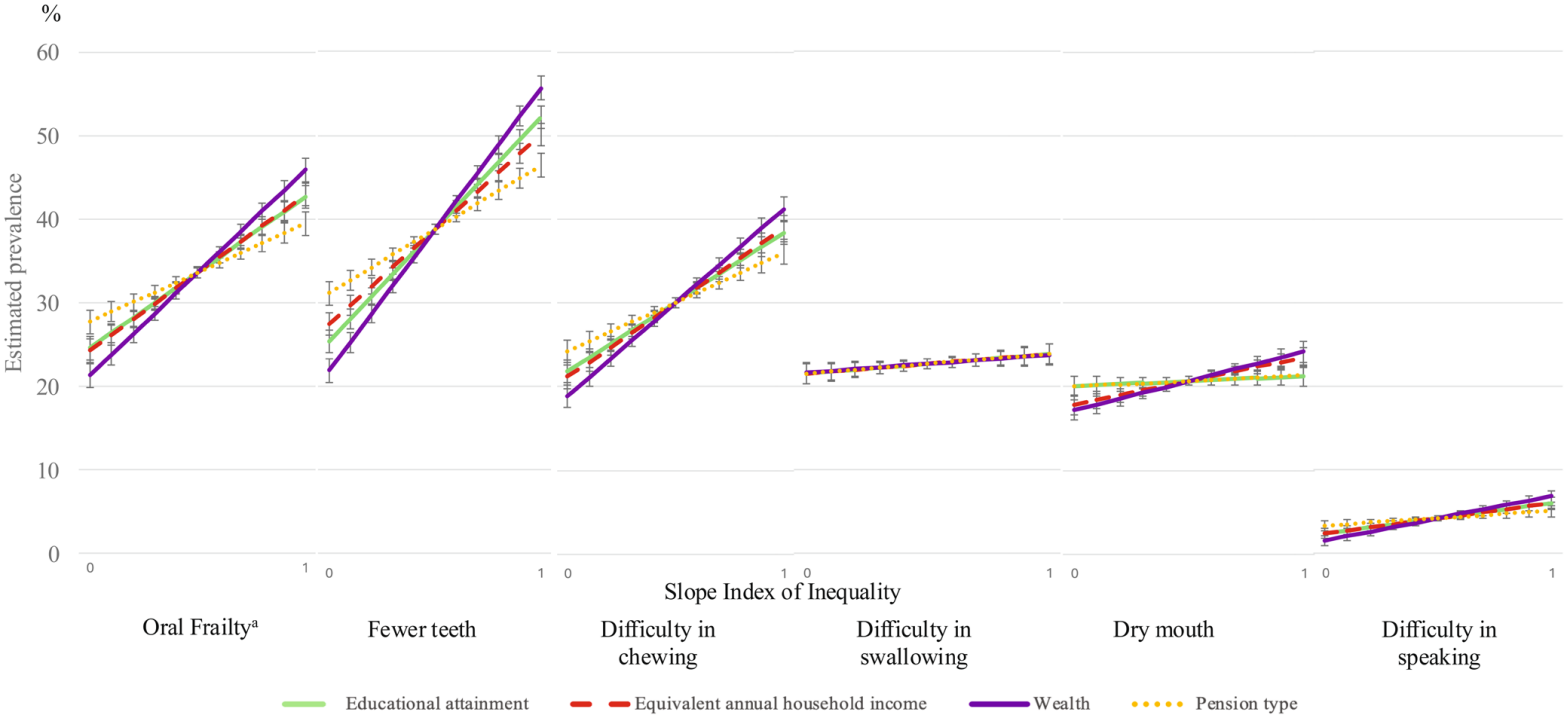
The Slope Index of Inequality illustrating the estimated prevalence of oral frailty, fewer teeth, difficulty in chewing, difficulty in swallowing, dry mouth, and difficulty in speaking by educational attainment, equivalent annual household income, wealth, and pension type after multiple imputation (*n* = 21,924). ^a^Oral frailty was defined by the presence of two or more of these components: fewer teeth, difficulty in chewing, difficulty in swallowing, dry mouth, and difficulty in speaking. Error bars represent the 95% confidence interval.

Figure 2 illustrates the SII based on the estimated prevalence of oral frailty and its components across four socioeconomic factors. The line plots were created using the SII values derived from the age‐ and sex‐adjusted models after multiple imputation, as shown in Table S12. The SII values were plotted on a standardised 0–1 socioeconomic rank scale, where 0 represents the most disadvantaged and 1 represents the most advantaged. This figure reveals clear inequality gradients for overall oral frailty. Regarding its components, fewer teeth and difficulty in chewing demonstrated the most pronounced socioeconomic inequalities. In contrast, inequalities for difficulty in swallowing, dry mouth, and difficulty in speaking were comparatively smaller.

To examine the extent to which each component contributes to the inequalities in oral frailty, we evaluated the changes in the SII when each component was individually added to the age‐ and sex‐adjusted models, as shown in Table S13. These results are also visually illustrated in Figures S3–S6 to enhance interpretability. Based on the percentage reduction in SII across the different adjusted models, the overall inequalities in oral frailty were largely explained by either fewer teeth or difficulty in chewing, with each independently accounting for approximately 50%–70% of the reduction in SII. In contrast, difficulty in swallowing, dry mouth, and difficulty in speaking contributed relatively less to the observed inequalities.

The results of the complete case analysis, as presented in Tables S14–S16 and Figure S7, were consistent with the findings obtained after MI, as shown in Tables 1 and 2, Table S13, and Figure 2, respectively. The RII also demonstrated consistent patterns with the SII across all analyses.

## 4 Discussion

To the best of our knowledge, this is the first study to comprehensively examine the association between SES and oral frailty. The novelty of this research lies in its incorporation of wealth and pension benefits—key indicators of SES among older adults—together with the evaluation of inequalities from both absolute and relative perspectives, and the application of SII and RII to enable comparative analysis across multiple components. Our findings revealed substantial socioeconomic inequalities in oral frailty and its components, even after adjusting for age and sex. Notably, wealth emerged as the most influential socioeconomic factor, demonstrating the largest SII and the largest RII in the fully adjusted model. These inequalities in oral frailty were primarily driven by disparities in tooth loss and chewing difficulties.

Our findings are consistent with previous research demonstrating a social gradient in other oral health outcomes. A systematic review and meta‐analysis concluded that lower SES was significantly associated with poorer oral health‐related quality of life among older adults (Baniasadi et al. 2021). In high‐income countries such as Japan and England, socioeconomic disparities persist among edentulous individuals, although Japan exhibits comparatively lower inequality—potentially due to its comprehensive dental care coverage (Ito et al. 2020). However, despite such coverage of dental prostheses, SES‐related inequalities in their usage remain among older Japanese adults (Matsuyama et al. 2014). Additionally, worse education and household expenditures have been linked to deteriorating oral health in older Japanese populations (Murakami et al. 2018). A Japanese study indicated that the number of remaining teeth (20 or more/19 or fewer natural teeth) significantly contributes to inequalities in oral frailty (Fujita et al. 2025). Complementing the above references, our study provides novel evidence highlighting the comprehensive socioeconomic inequalities in oral frailty among older individuals in Japan.

According to a systematic review, wealth represents accumulated resources over a lifetime, making it a more consistent and influential determinant of health inequalities than income or education (Acciai 2018; Pollack et al. 2007). This stability may explain why wealth emerged as the most critical socioeconomic factor contributing to inequalities in oral frailty in our study. On another concern, tooth loss and chewing difficulties were the primary drivers of these inequalities, likely due to their substantial impacts on nutrition, eating ability, and overall quality of life among older adults (Atanda et al. 2022). These conditions have immediate and tangible effects on daily functioning (Dibello et al. 2023). While challenges such as swallowing difficulties, dry mouth, and speaking difficulties also cause discomfort and problems for older adults, they may be more manageable through practical, self‐directed strategies and thus exert less influence on inequality (Lipsky et al. 2024). For instance, hydration and softer foods may ease swallowing issues (Reber et al. 2019), dry mouth can be alleviated with salivary substitutes (Lipsky et al. 2024), and speaking difficulties can be improved through assistive devices (Sigafoos et al. 2014). In contrast, tooth loss and chewing difficulties are considerably more difficult to address, which may explain their dominant role in shaping oral frailty disparities.

The association between socioeconomic inequalities and oral frailty may be attributed to multiple interrelated mechanisms. Individuals with lower SES often encounter considerable barriers to accessing preventive dental care, such as limited insurance coverage, fewer dental visits, and inadequate oral hygiene practices (Locker et al. 2011; Morohoshi et al. 2024; Oberoi et al. 2016). These challenges contribute to delays in seeking treatment and hinder disease prevention efforts. Furthermore, lower SES is frequently associated with unhealthy behaviours and poor nutritional intake, which can accelerate the progression of oral diseases (Moynihan and Petersen 2004). Chronic stress and systemic inflammation, both prevalent among individuals with lower SES, may also exacerbate periodontal deterioration (Peruzzo et al. 2007). Collectively, these socioeconomic factors can result in cumulative oral health damage, including tooth loss and masticatory difficulties, thereby increasing the risk of oral frailty. While these mechanisms offer plausible explanations, this study focused on examining the socioeconomic distribution of oral frailty and its components, rather than exploring the underlying causes of these inequalities.

This study has important public health implications. It contributes to suggesting disadvantaged populations who may be prone to oral frailty among older adults. If not adequately managed, oral frailty can worsen, leading to a decline in oral function and eventually disability of oral function, which is associated with multiple serious health issues such as frailty or neurodegenerative diseases (Tanaka et al. 2024). Early detection of oral frailty can help vulnerable populations engage in interventions that effectively improve oral health functions through targeted oral frailty measures (Shirobe et al. 2022). Our findings may provide useful insights for policymakers when designing targeted interventions for older adults with limited socioeconomic conditions, which could contribute to reducing oral health inequalities. Public health efforts should focus on individuals with lower education, income, wealth, and pension benefits through accessible oral health education, screening, and preventive care. In Japan, treatment for oral hypofunction—a clinical concept closely related to oral frailty—has been covered by national health insurance since 2018 (Japan Dental Association 2019; Sato et al. 2021). Therefore, promoting the utilisation of this treatment may offer a practical strategy to support socioeconomically disadvantaged older adults.

This study has several strengths. First, the robust study design and application of advanced inequality measures (SII and RII) enabled a detailed and comparative analysis of socioeconomic disparities. Second, the relatively large sample size derived from the well‐established JAGES database enhanced statistical power and generalisability, strengthening the validity of our conclusions.

However, there are several limitations to consider. First, as this was a cross‐sectional study, it was not possible to definitively determine the direction of causality between socioeconomic factors and oral frailty. A future longitudinal study is needed to confirm the temporality of this association. Second, the variables related to oral frailty and SES were obtained from self‐reported questionnaires. This may introduce information bias, especially differential misclassification across socioeconomic groups. Therefore, our findings should be interpreted with caution, considering the possibility of socioeconomic differences in reporting accuracy. Third, due to the unavailability of data, this study employed a modified version of the OF‐5, in which the fifth component, ‘low articulatory oral motor skills’, was replaced with ‘difficulty in speaking’. This modification may have affected the accuracy of the oral frailty assessment. Indeed, the prevalence of oral frailty in our study (33.6%) was slightly lower than that reported in previous studies (36.7% and 39.3%) (Iwasaki et al. 2024; Tanaka et al. 2024), suggesting that our results may underestimate the true prevalence.

In conclusion, substantial socioeconomic inequalities in oral frailty and its components among older adults were identified, with wealth playing the most crucial role. Among the components, disparities in tooth loss and chewing difficulties contributed most significantly to the observed inequalities.

## Author Contributions

**Duc Sy Minh Ho:** conceptualization, methodology, software, writing – review and editing, writing – original draft, formal analysis, visualization. **Yusuke Matsuyama:** conceptualization, investigation, writing – review and editing. **Sakura Kiuchi:** conceptualization, investigation, writing – review and editing. **Tatsuo Yamamoto:** conceptualization, investigation, writing – review and editing. **Jun Aida:** conceptualization, methodology, formal analysis, writing – review and editing, funding acquisition, supervision, investigation.

## Funding

This study was supported by the FUTOKUKAI Foundation, Grant‐in‐Aid for Scientific Research (22K10285, 23K27807, 23K09500, 24K02658, 20H00557, 20K10540, 21H03196, 21K17302, 22H00934, 22H03299, 22K04450, 22K13558, 22K17409, 23H00449, 23K21500, 19H03860, 19H03861, 21K19635) from the Japan Society for the Promotion of Science (JSPS), Health Labour Sciences Research Grants (19FA1012, 19FA2001, 21FA1012, 22FA2001, 22FA1010, 22FG2001, 23FA1022, 25FA1013), Research Institute of Science and Technology for Society (JPMJOP1831) from the Japan Science and Technology (JST), a grant from Japan Health Promotion and Fitness Foundation, and National Research Institute for Earth Science and Disaster Resilience.

## Ethics Statement

All procedures involving human participants in this study were conducted in accordance with the Declaration of Helsinki. Ethical approval for the secondary data analysis was granted by the Ethics Review Committee of Tokyo Medical and Dental University, Japan (Approval No. D2022‐040). JAGES was approved by the Ethics Committee on the Research of Human Subjects of Chiba University (Approval No. 2493) and the National Center for Geriatrics and Gerontology, Japan (Approval No. 992‐3). The data were used with the participants' consent.

## Conflicts of Interest

The authors declare no conflicts of interest.

## Supporting information


**Table S1:** Numbers and percentages of missing variables used in multiple imputation (*n* = 21,924).
**Table S2:** Demographic characteristics of participants, stratified by the prevalence of fewer teeth after multiple imputation (*n* = 21,924).
**Table S3:** Demographic characteristics of participants, stratified by the prevalence of difficulty in chewing after multiple imputation (*n* = 21,924).
**Table S4:** Demographic characteristics of participants, stratified by the prevalence of difficulty in swallowing after multiple imputation (*n* = 21,924).
**Table S5:** Demographic characteristics of participants, stratified by the prevalence of dry mouth after multiple imputation (*n* = 21,924).
**Table S6:** Demographic characteristics of participants, stratified by the prevalence of difficulty in speaking after multiple imputation (*n* = 21,924).
**Table S7:** The Slope Index of Inequality (SII) and Relative Index of Inequality (RII) of socioeconomic factors in the prevalence of fewer teeth after multiple imputation (*n* = 21,924).
**Table S8:** The Slope Index of Inequality (SII) and Relative Index of Inequality (RII) of socioeconomic factors in the prevalence of difficulty in chewing after multiple imputation (*n* = 21,924).
**Table S9:** The Slope Index of Inequality (SII) and Relative Index of Inequality (RII) of socioeconomic factors in the prevalence of difficulty in swallowing after multiple imputation (*n* = 21,924).
**Table S10:** The Slope Index of Inequality (SII) and Relative Index of Inequality (RII) of socioeconomic factors in the prevalence of dry mouth after multiple imputation (*n* = 21,924).
**Table S11:** The Slope Index of Inequality (SII) and Relative Index of Inequality (RII) of socioeconomic factors in the prevalence of difficulty in speaking after multiple imputation (*n* = 21,924).
**Table S12:** The Slope Index of Inequality (SII) of socioeconomic factors in the prevalence of oral frailty and its components after multiple imputation (*n* = 21,924).
**Table S13:** Changes in the Slope Index of Inequality (SII) when each component of oral frailty was separately added to the age‐ and sex‐adjusted models after multiple imputation (*n* = 21,924).
**Table S14:** Demographic characteristics of participants, stratified by the prevalence of oral frailty by complete case analysis (*n* = 15,114).
**Table S15:** The Slope Index of Inequality (SII) and Relative Index of Inequality (RII) of socioeconomic factors in the prevalence of oral frailty by complete case analysis (*n* = 15,114).
**Table S16:** Changes in the Slope Index of Inequality (SII) when each component of oral frailty was separately added to the age‐ and sex‐adjusted models by complete case analysis (*n* = 15,114).
**Figure S1:** Flowchart of the study participants.
**Figure S2:** Theoretical framework using directed acyclic graph (DAG) for the socioeconomic inequalities in oral frailty and its components including confounders (age and sex).
**Figure S3:** Contribution of inequalities in each oral frailty component to the overall inequality in oral frailty, by educational attainment (*n* = 21,924).
**Figure S4:** Contribution of inequalities in each oral frailty component to the overall inequality in oral frailty, by equivalent annual household income (*n* = 21,924).
**Figure S5:** Contribution of inequalities in each oral frailty component to the overall inequality in oral frailty, by wealth (*n* = 21,924).
**Figure S6:** Contribution of inequalities in each oral frailty component to the overall inequality in oral frailty, by pension type (*n* = 21,924).
**Figure S7:** The Slope Index of Inequality illustrating the estimated prevalence of oral frailty, fewer teeth, difficulty in chewing, difficulty in swallowing, dry mouth, and difficulty in speaking by educational attainment, equivalent annual household income, wealth, and pension type by complete case analysis (*n* = 15,114).

## Data Availability

The datasets used in this research are available from the corresponding author upon reasonable request. All inquiries should be directed to the data management committee via email at dataadmin.ml@jages.net.
