# Supplementary material for: Socioeconomic Inequalities in Oral Frailty and Its Components
Source: Oral Dis. 2025 Dec 16;32(6):1709–17. doi: 10.1111/odi.70169 (PMC13457687; doi:10.1111/odi.70169)
Supplement: Supplementary file 1 [file ODI-32-1709-s001.docx]

**Supplementary Material**

**Socioeconomic inequalities in oral frailty and its components**

**Supplementary Table Legend**

Supplementary Table 1. Numbers and percentages of missing variables used in multiple imputation (n = 21,924)

Supplementary Table 2. Demographic characteristics of participants, stratified by the prevalence of fewer teeth after multiple imputation (n = 21,924)

Supplementary Table 3. Demographic characteristics of participants, stratified by the prevalence of difficulty in chewing after multiple imputation (n = 21,924)

Supplementary Table 4. Demographic characteristics of participants, stratified by the prevalence of difficulty in swallowing after multiple imputation (n = 21,924)

Supplementary Table 5. Demographic characteristics of participants, stratified by the prevalence of dry mouth after multiple imputation (n = 21,924)

Supplementary Table 6. Demographic characteristics of participants, stratified by the prevalence of difficulty in speaking after multiple imputation (n = 21,924)

Supplementary Table 7. The Slope Index of Inequality (SII) and Relative Index of Inequality (RII) of socioeconomic factors in the prevalence of fewer teeth after multiple imputation (n = 21,924)

Supplementary Table 8. The Slope Index of Inequality (SII) and Relative Index of Inequality (RII) of socioeconomic factors in the prevalence of difficulty in chewing after multiple imputation (n = 21,924)

Supplementary Table 9. The Slope Index of Inequality (SII) and Relative Index of Inequality (RII) of socioeconomic factors in the prevalence of difficulty in swallowing after multiple imputation (n = 21,924)

Supplementary Table 10. The Slope Index of Inequality (SII) and Relative Index of Inequality (RII) of socioeconomic factors in the prevalence of dry mouth after multiple imputation (n = 21,924)

Supplementary Table 11. The Slope Index of Inequality (SII) and Relative Index of Inequality (RII) of socioeconomic factors in the prevalence of difficulty in speaking after multiple imputation (n = 21,924)

Supplementary Table 12. The Slope Index of Inequality (SII) of socioeconomic factors in the prevalence of oral frailty and its components after multiple imputation (n = 21,924)

Supplementary Table 13. Changes in the Slope Index of Inequality (SII) when each component of oral frailty was separately added to the age- and sex-adjusted models after multiple imputation (n = 21,924)

Supplementary Table 14. Demographic characteristics of participants, stratified by the prevalence of oral frailty by complete case analysis (n = 15,114)

Supplementary Table 15. The Slope Index of Inequality (SII) and Relative Index of Inequality (RII) of socioeconomic factors in the prevalence of oral frailty by complete case analysis (n = 15,114)

Supplementary Table 16. Changes in the Slope Index of Inequality (SII) when each component of oral frailty was separately added to the age- and sex-adjusted models by complete case analysis (n = 15,114)

Supplementary Table 1. Numbers and percentages of missing variables used in multiple imputation (n = 21,924)

| **Variables** | **Number and percentage**  **of missing values** | |
| --- | --- | --- |
|  | **n** | **%** |
| Age | 0 | 0 |
| Sex | 0 | 0 |
| Educational attainment | 594 | 2.71 |
| Equivalent annual household income | 2,904 | 13.25 |
| Wealth | 4,257 | 19.42 |
| Pension | 580 | 2.65 |
| Fewer teeth | 508 | 2.32 |
| Difficulty in chewing | 380 | 1.73 |
| Difficulty in swallowing | 407 | 1.86 |
| Dry mouth | 511 | 2.33 |
| Difficulty in speaking | 2,225 | 10.15 |
| Oral frailty^a^ | 3,074 | 14.02 |

^a^: Oral frailty was defined by the presence of two or more of these components: fewer teeth, difficulty in chewing, difficulty in swallowing, dry mouth, and difficulty in speaking

Supplementary Table 2. Demographic characteristics of participants, stratified by the prevalence of fewer teeth after multiple imputation (n = 21,924)

| **Characteristics** | **Fewer Teeth** | | |
| --- | --- | --- | --- |
|  | **Absence** | **Presence** | **Overall** |
|  | **(row%^a^)** | **(row%^a^)** | **No. (col%^b^)** |
|  | **(n = 13,420)** | **(n = 8,504)** | **(n = 21,924)** |
| **Age** |  |  |  |
| 65-69 years | 74.8 | 25.2 | 5,088 (23.2%) |
| 70-74 years | 65.2 | 34.8 | 6,542 (29.8%) |
| 75-79 years | 58.1 | 41.9 | 5,032 (23.0%) |
| 80-84 years | 50.4 | 49.6 | 3,495 (15.9%) |
| ≥85 years | 37.6 | 62.4 | 1,767 (8.1%) |
| **Sex** |  |  |  |
| Male | 59.2 | 40.8 | 10,459 (47.7%) |
| Female | 63.0 | 37.0 | 11,465 (52.3%) |
| **Educational attainment** |  |  |  |
| ≥13 years | 70.5 | 29.5 | 7,588 (34.6%) |
| 10-12 years | 61.8 | 38.2 | 9,667 (44.1%) |
| ≤9 years | 44.9 | 55.1 | 4,669 (21.3%) |
| **Equivalent annual household income** |  |  |  |
| ≥4.0 million yen | 70.1 | 29.9 | 2,582 (11.8%) |
| 3-3.99 million yen | 68.2 | 31.8 | 3,241 (14.8%) |
| 2-2.99 million yen | 66.8 | 33.2 | 5,132 (23.4%) |
| 1-1.99 million yen | 57.7 | 42.3 | 7,983 (36.4%) |
| <1 million yen | 45.6 | 54.4 | 2,986 (13.6%) |
| **Wealth** |  |  |  |
| ≥50 million yen | 73.8 | 26.2 | 3,024 (13.8%) |
| 10-49.9 million yen | 67.4 | 32.6 | 9,123 (41.6%) |
| 5-9.99 million yen | 58.8 | 41.2 | 3,636 (16.6%) |
| 1-4.99 million yen | 51.4 | 48.6 | 3,764 (17.2%) |
| <1 million yen | 41.0 | 59.0 | 2,376 (10.8%) |
| **Pension type** |  |  |  |
| Rich^c^ | 69.2 | 30.8 | 4,279 (19.5%) |
| Moderate^d^ | 61.2 | 38.8 | 12,642 (57.7%) |
| Poor^e^ | 54.7 | 45.3 | 4,758 (21.7%) |
| None^f^ | 50.8 | 49.2 | 245 (1.1%) |
| ^a^: Row percentage  ^b^: Column percentage  ^c^: Enrolled in Corporate Pension Plan or Personal Pension Plan, either with or without National Pension Plan, Employees' Pension Plan or Mutual-Aid Pension Plan | | | |
| ^d^: Enrolled in Employees' Pension Plan or Mutual-Aid Pension Plan, either with or without National Pension Plan  ^e^: Enrolled in National Pension Plan  ^f^: No pension benefits | | | |

Supplementary Table 3. Demographic characteristics of participants, stratified by the prevalence of difficulty in chewing after multiple imputation (n = 21,924)

| **Characteristics** | **Difficulty in Chewing** | | |
| --- | --- | --- | --- |
|  | **Absence** | **Presence** | **Overall** |
|  | **(row%^a^)** | **(row%^a^)** | **No. (col%^b^)** |
|  | **(n = 15,327)** | **(n = 6,597)** | **(n = 21,924)** |
| **Age** |  |  |  |
| 65-69 years | 76.2 | 23.8 | 5,088 (23.2%) |
| 70-74 years | 71.4 | 28.6 | 6,542 (29.8%) |
| 75-79 years | 68.8 | 31.2 | 5,032 (23.0%) |
| 80-84 years | 65.2 | 34.8 | 3,495 (15.9%) |
| ≥85 years | 58.9 | 41.1 | 1,767 (8.1%) |
| **Sex** |  |  |  |
| Male | 69.3 | 30.7 | 10,459 (47.7%) |
| Female | 70.5 | 29.5 | 11,465 (52.3%) |
| **Educational attainment** |  |  |  |
| ≥13 years | 75.6 | 24.4 | 7,588 (34.6%) |
| 10 -12 years | 69.8 | 30.2 | 9,667 (44.1%) |
| ≤9 years | 60.9 | 39.1 | 4,669 (21.3%) |
| **Equivalent annual household income** |  |  |  |
| ≥4.0 million yen | 78.1 | 21.9 | 2,582 (11.8%) |
| 3-3.99 million yen | 74.7 | 25.3 | 3,241 (14.8%) |
| 2-2.99 million yen | 73.0 | 27.0 | 5,132 (23.4%) |
| 1-1.99 million yen | 67.2 | 32.8 | 7,983 (36.4%) |
| <1 million yen | 59.6 | 40.4 | 2,986 (13.6%) |
| **Wealth** |  |  |  |
| ≥50 million yen | 79.2 | 20.8 | 3,024 (13.8%) |
| 10-49.9 million yen | 73.4 | 26.6 | 9,123 (41.6%) |
| 5-9.99 million yen | 68.2 | 31.8 | 3,636 (16.6%) |
| 1-4.99 million yen | 63.8 | 36.2 | 3,764 (17.2%) |
| <1 million yen | 57.0 | 43.0 | 2,376 (10.8%) |
| **Pension type** |  |  |  |
| Rich^c^ | 75.3 | 24.7 | 4,279 (19.5%) |
| Moderate^d^ | 70.0 | 30.0 | 12,642 (57.7%) |
| Poor^e^ | 65.4 | 34.6 | 4,758 (21.7%) |
| None^f^ | 57.9 | 42.1 | 245 (1.1%) |
| ^a^: Row percentage  ^b^: Column percentage  ^c^: Enrolled in Corporate Pension Plan or Personal Pension Plan, either with or without National Pension Plan, Employees' Pension Plan or Mutual-Aid Pension Plan | | | |
| ^d^: Enrolled in Employees' Pension Plan or Mutual-Aid Pension Plan, either with or without National Pension Plan  ^e^: Enrolled in National Pension Plan  ^f^: No pension benefits | | | |

Supplementary Table 4. Demographic characteristics of participants, stratified by the prevalence of difficulty in swallowing after multiple imputation (n = 21,924)

| **Characteristics** | **Difficulty in Swallowing** | | |
| --- | --- | --- | --- |
|  | **Absence** | **Presence** | **Overall** |
|  | **(row%^a^)** | **(row%^a^)** | **No. (col%^b^)** |
|  | **(n = 16,949)** | **(n = 4,975)** | **(n = 21,924)** |
| **Age** |  |  |  |
| 65-69 years | 80.9 | 19.1 | 5,088 (23.2%) |
| 70-74 years | 77.8 | 22.2 | 6,542 (29.8%) |
| 75-79 years | 75.6 | 24.4 | 5,032 (23.0%) |
| 80-84 years | 76.1 | 23.9 | 3,495 (15.9%) |
| ≥85 years | 72.8 | 27.2 | 1,767 (8.1%) |
| **Sex** |  |  |  |
| Male | 77.6 | 22.4 | 10,459 (47.7%) |
| Female | 77.0 | 23.0 | 11,465 (52.3%) |
| **Educational attainment** |  |  |  |
| ≥13 years | 78.5 | 21.5 | 7,588 (34.6%) |
| 10 -12 years | 77.3 | 22.7 | 9,667 (44.1%) |
| ≤9 years | 75.4 | 24.6 | 4,669 (21.3%) |
| **Equivalent annual household income** |  |  |  |
| ≥4.0 million yen | 79.1 | 20.9 | 2,582 (11.8%) |
| 3-3.99 million yen | 78.8 | 21.2 | 3,241 (14.8%) |
| 2-2.99 million yen | 76.8 | 23.2 | 5,132 (23.4%) |
| 1-1.99 million yen | 77.3 | 22.7 | 7,983 (36.4%) |
| <1 million yen | 75.0 | 25.0 | 2,986 (13.6%) |
| **Wealth** |  |  |  |
| ≥50 million yen | 78.1 | 21.9 | 3,024 (13.8%) |
| 10-49.9 million yen | 77.8 | 22.2 | 9,123 (41.6%) |
| 5-9.99 million yen | 77.6 | 22.4 | 3,636 (16.6%) |
| 1-4.99 million yen | 75.9 | 24.1 | 3,764 (17.2%) |
| <1 million yen | 76.3 | 23.7 | 2,376 (10.8%) |
| **Pension type** |  |  |  |
| Rich^c^ | 78.6 | 21.4 | 4,279 (19.5%) |
| Moderate^d^ | 77.5 | 22.5 | 12,642 (57.7%) |
| Poor^e^ | 75.7 | 24.3 | 4,758 (21.7%) |
| None^f^ | 75.9 | 24.1 | 245 (1.1%) |
| ^a^: Row percentage  ^b^: Column percentage  ^c^: Enrolled in Corporate Pension Plan or Personal Pension Plan, either with or without National Pension Plan, Employees' Pension Plan or Mutual-Aid Pension Plan | | | |
| ^d^: Enrolled in Employees' Pension Plan or Mutual-Aid Pension Plan, either with or without National Pension Plan  ^e^: Enrolled in National Pension Plan  ^f^: No pension benefits | | | |

Supplementary Table 5. Demographic characteristics of participants, stratified by the prevalence of dry mouth after multiple imputation (n = 21,924)

| **Characteristics** | **Dry Mouth** | | |
| --- | --- | --- | --- |
|  | **Absence** | **Presence** | **Overall** |
|  | **(row%^a^)** | **(row%^a^)** | **No. (col%^b^)** |
|  | **(n = 17,391)** | **(n = 4,533)** | **(n = 21,924)** |
| **Age** |  |  |  |
| 65-69 years | 80.9 | 19.1 | 5,088 (23.2%) |
| 70-74 years | 80.6 | 19.4 | 6,542 (29.8%) |
| 75-79 years | 77.8 | 22.2 | 5,032 (23.0%) |
| 80-84 years | 77.7 | 22.3 | 3,495 (15.9%) |
| ≥85 years | 77.5 | 22.5 | 1,767 (8.1%) |
| **Sex** |  |  |  |
| Male | 80.0 | 20.0 | 10,459 (47.7%) |
| Female | 78.7 | 21.3 | 11,465 (52.3%) |
| **Educational attainment** |  |  |  |
| ≥13 years | 79.7 | 20.3 | 7,588 (34.6%) |
| 10-12 years | 79.9 | 20.1 | 9,667 (44.1%) |
| ≤9 years | 77.5 | 22.5 | 4,669 (21.3%) |
| **Equivalent annual household income** |  |  |  |
| ≥4.0 million yen | 82.2 | 17.8 | 2,582 (11.8%) |
| 3-3.99 million yen | 81.5 | 18.5 | 3,241 (14.8%) |
| 2-2.99 million yen | 79.9 | 20.1 | 5,132 (23.4%) |
| 1-1.99 million yen | 78.3 | 21.7 | 7,983 (36.4%) |
| <1 million yen | 76.3 | 23.7 | 2,986 (13.6%) |
| **Wealth** |  |  |  |
| ≥50 million yen | 82.7 | 17.3 | 3,024 (13.8%) |
| 10-49.9 million yen | 80.3 | 19.7 | 9,123 (41.6%) |
| 5-9.99 million yen | 78.4 | 21.6 | 3,636 (16.6%) |
| 1-4.99 million yen | 77.8 | 22.2 | 3,764 (17.2%) |
| <1 million yen | 75.4 | 24.6 | 2,376 (10.8%) |
| **Pension type** |  |  |  |
| Rich^c^ | 80.9 | 19.1 | 4,279 (19.5%) |
| Moderate^d^ | 79.0 | 21.0 | 12,642 (57.7%) |
| Poor^e^ | 78.9 | 21.1 | 4,758 (21.7%) |
| None^f^ | 77.7 | 22.3 | 245 (1.1%) |
| ^a^: Row percentage  ^b^: Column percentage  ^c^: Enrolled in Corporate Pension Plan or Personal Pension Plan, either with or without National Pension Plan, Employees' Pension Plan or Mutual-Aid Pension Plan | | | |
| ^d^: Enrolled in Employees' Pension Plan or Mutual-Aid Pension Plan, either with or without National Pension Plan  ^e^: Enrolled in National Pension Plan  ^f^: No pension benefits | | | |

Supplementary Table 6. Demographic characteristics of participants, stratified by the prevalence of difficulty in speaking after multiple imputation (n=21,924)

| **Characteristics** | **Difficulty in Speaking** | | |
| --- | --- | --- | --- |
|  | **Absence** | **Presence** | **Overall** |
|  | **(row%^a^)** | **(row%^a^)** | **No. (col%^b^)** |
|  | **(n = 20,994)** | **(n = 930)** | **(n = 21,924)** |
| **Age** |  |  |  |
| 65-69 years | 96.7 | 3.3 | 5,088 (23.2%) |
| 70-74 years | 95.7 | 4.3 | 6,542 (29.8%) |
| 75-79 years | 95.3 | 4.7 | 5,032 (23.0%) |
| 80-84 years | 95.4 | 4.6 | 3,495 (15.9%) |
| ≥85 years | 95.1 | 4.9 | 1,767 (8.1%) |
| **Sex** |  |  |  |
| Male | 94.8 | 5.2 | 10,459 (47.7%) |
| Female | 96.6 | 3.4 | 11,465 (52.3%) |
| **Educational attainment** |  |  |  |
| ≥13 years | 96.8 | 3.2 | 7,588 (34.6%) |
| 10-12 years | 95.7 | 4.3 | 9,667 (44.1%) |
| ≤9 years | 94.1 | 5.9 | 4,669 (21.3%) |
| **Equivalent annual household income** |  |  |  |
| ≥4.0 million yen | 96.9 | 3.1 | 2,582 (11.8%) |
| 3-3.99 million yen | 96.6 | 3.4 | 3,241 (14.8%) |
| 2-2.99 million yen | 96.3 | 3.7 | 5,132 (23.4%) |
| 1-1.99 million yen | 95.6 | 4.4 | 7,983 (36.4%) |
| <1 million yen | 93.2 | 6.8 | 2,986 (13.6%) |
| **Wealth** |  |  |  |
| ≥50 million yen | 97.4 | 2.6 | 3,024 (13.8%) |
| 10-49.9 million yen | 96.7 | 3.3 | 9,123 (41.6%) |
| 5-9.99 million yen | 95.7 | 4.3 | 3,636 (16.6%) |
| 1-4.99 million yen | 94.2 | 5.8 | 3,764 (17.2%) |
| <1 million yen | 92.6 | 7.4 | 2,376 (10.8%) |
| **Pension type** |  |  |  |
| Rich^c^ | 96.6 | 3.4 | 4,279 (19.5%) |
| Moderate^d^ | 95.5 | 4.5 | 12,642 (57.7%) |
| Poor^e^ | 95.7 | 4.3 | 4,758 (21.7%) |
| None^f^ | 94.3 | 5.7 | 245 (1.1%) |
| ^a^: Row percentage  ^b^: Column percentage  ^c^: Enrolled in Corporate Pension Plan or Personal Pension Plan, either with or without National Pension Plan, Employees' Pension Plan or Mutual-Aid Pension Plan | | | |
| ^d^: Enrolled in Employees' Pension Plan or Mutual-Aid Pension Plan, either with or without National Pension Plan  ^e^: Enrolled in National Pension Plan  ^f^: No pension benefits | | | |

Supplementary Table 7. The Slope Index of Inequality (SII) and Relative Index of Inequality (RII) of socioeconomic factors in the prevalence of fewer teeth after multiple imputation (n = 21,924)

|  | **Age- and sex-adjusted model^a^** | | | | **Fully-adjusted model^b^** | | | |
| --- | --- | --- | --- | --- | --- | --- | --- | --- |
|  | **SII^c^** |  | **RII^d^** |  | **SII** |  | **RII** |  |
|  | 95% CI^e^ | P value | 95% CI | P value | 95% CI | P value | 95% CI | P value |
| Educational attainment | 0.27 | <0.01 | 1.92 | <0.01 | 0.17 | <0.01 | 1.47 | <0.01 |
|  | (0.24; 0.29) |  | (1.80; 2.04) |  | (0.14; 0.19) |  | (1.38; 1.57) |  |
| Equivalent annual household income | 0.23 | <0.01 | 1.81 | <0.01 | 0.06 | <0.01 | 1.19 | <0.01 |
|  | (0.20; 0.25) |  | (1.70; 1.94) |  | (0.03; 0.09) |  | (1.10; 1.28) |  |
| Wealth | 0.34 | <0.01 | 2.28 | <0.01 | 0.25 | <0.01 | 1.83 | <0.01 |
|  | (0.31; 0.36) |  | (2.15; 2.43) |  | (0.23; 0.28) |  | (1.70; 1.96) |  |
| Pension type | 0.15 | <0.01 | 1.49 | <0.01 | 0.07 | <0.01 | 1.18 | <0.01 |
|  | (0.13; 0.18) |  | (1.39; 1.59) |  | (0.04; 0.09) |  | (1.10; 1.26) |  |

^a^: Adjusted for age and sex. Each socioeconomic factor was separately included

^b^: Adjusted for age, sex and other socioeconomic factors

^c^: Slope Index of Inequality

^d^: Relative Index of Inequality

^e^: Confidence Interval

Supplementary Table 8. The Slope Index of Inequality (SII) and Relative Index of Inequality (RII) of socioeconomic factors in the prevalence of difficulty in chewing after multiple imputation (n = 21,924)

|  | **Age- and sex-adjusted model^a^** | | | | **Fully-adjusted model^b^** | | | |
| --- | --- | --- | --- | --- | --- | --- | --- | --- |
|  | **SII^c^** |  | **RII^d^** |  | **SII** |  | **RII** |  |
|  | 95% CI^e^ | P value | 95% CI | P value | 95% CI | P value | 95% CI | P value |
| Educational attainment | 0.17 | <0.01 | 1.73 | <0.01 | 0.10 | <0.01 | 1.35 | <0.01 |
|  | (0.14; 0.19) |  | (1.60; 1.87) |  | (0.07; 0.12) |  | (1.25; 1.47) |  |
| Equivalent annual household income | 0.18 | <0.01 | 1.82 | <0.01 | 0.08 | <0.01 | 1.28 | <0.01 |
|  | (0.15; 0.20) |  | (1.67; 1.99) |  | (0.05; 0.11) |  | (1.16; 1.42) |  |
| Wealth | 0.22 | <0.01 | 2.07 | <0.01 | 0.15 | <0.01 | 1.65 | <0.01 |
|  | (0.20; 0.25) |  | (1.91; 2.25) |  | (0.12; 0.18) |  | (1.49; 1.82) |  |
| Pension type | 0.12 | <0.01 | 1.49 | <0.01 | 0.06 | <0.01 | 1.19 | <0.01 |
|  | (0.09; 0.14) |  | (1.37; 1.61) |  | (0.03; 0.08) |  | (1.10; 1.30) |  |

^a^: Adjusted for age and sex. Each socioeconomic factor was separately included

^b^: Adjusted for age, sex and other socioeconomic factors

^c^: Slope Index of Inequality

^d^: Relative Index of Inequality

^e^: Confidence Interval

Supplementary Table 9. The Slope Index of Inequality (SII) and Relative Index of Inequality (RII) of socioeconomic factors in the prevalence of difficulty in swallowing after multiple imputation (n = 21,924)

|  | **Age- and sex-adjusted model^a^** | | | | **Fully-adjusted model^b^** | | | |
| --- | --- | --- | --- | --- | --- | --- | --- | --- |
|  | **SII^c^** |  | **RII^d^** |  | **SII** |  | **RII** |  |
|  | 95% CI^e^ | P value | 95% CI | P value | 95% CI | P value | 95% CI | P value |
| Educational attainment | 0.02 | <0.05 | 1.11 | <0.05 | 0.02 | >0.05 | 1.07 | >0.05 |
|  | (0.001; 0.05) |  | (1.004; 1.22) |  | (-0.01; 0.04) |  | (0.97; 1.19) |  |
| Equivalent annual household income | 0.02 | <0.05 | 1.10 | <0.05 | 0.01 | >0.05 | 1.05 | >0.05 |
|  | (0.002; 0.05) |  | (1.002; 1.21) |  | (-0.01; 0.04) |  | (0.94; 1.18) |  |
| Wealth | 0.02 | >0.05 | 1.10 | >0.05 | 0.01 | >0.05 | 1.04 | >0.05 |
|  | (-0.0004; 0.04) |  | (0.998; 1.21) |  | (-0.02; 0.03) |  | (0.93; 1.17) |  |
| Pension type | 0.02 | <0.05 | 1.11 | <0.05 | 0.02 | >0.05 | 1.08 | >0.05 |
|  | (0.001; 0.05) |  | (1.001; 1.23) |  | (-0.01; 0.04) |  | (0.97; 1.19) |  |

^a^: Adjusted for age and sex. Each socioeconomic factor was separately included

^b^: Adjusted for age, sex and other socioeconomic factors

^c^: Slope Index of Inequality

^d^: Relative Index of Inequality

^e^: Confidence Interval

Supplementary Table 10. The Slope Index of Inequality (SII) and Relative Index of Inequality (RII) of socioeconomic factors in the prevalence of dry mouth after multiple imputation (n = 21,924)

|  | **Age- and sex-adjusted model^a^** | | | | **Fully-adjusted model^b^** | | | |
| --- | --- | --- | --- | --- | --- | --- | --- | --- |
|  | **SII^c^** |  | **RII^d^** |  | **SII** |  | **RII** |  |
|  | 95% CI^e^ | P value | 95% CI | P value | 95% CI | P value | 95% CI | P value |
| Educational attainment | 0.01 | >0.05 | 1.05 | >0.05 | -0.01 | >0.05 | 0.94 | >0.05 |
|  | (-0.01; 0.03) |  | (0.95; 1.17) |  | (-0.04; 0.01) |  | (0.85; 1.05) |  |
| Equivalent annual household income | 0.06 | <0.01 | 1.31 | <0.01 | 0.03 | <0.05 | 1.17 | <0.05 |
|  | (0.04; 0.08) |  | (1.18; 1.46) |  | (0.01; 0.06) |  | (1.04; 1.32) |  |
| Wealth | 0.07 | <0.01 | 1.39 | <0.01 | 0.06 | <0.01 | 1.32 | <0.01 |
|  | (0.05; 0.09) |  | (1.25; 1.56) |  | (0.03; 0.09) |  | (1.16; 1.51) |  |
| Pension type | 0.01 | >0.05 | 1.06 | >0.05 | -0.004 | >0.05 | 0.97 | >0.05 |
|  | (-0.01; 0.04) |  | (0.95; 1.17) |  | (-0.03; 0.02) |  | (0.87; 1.08) |  |

^a^: Adjusted for age and sex. Each socioeconomic factor was separately included

^b^: Adjusted for age, sex and other socioeconomic factors

^c^: Slope Index of Inequality

^d^: Relative Index of Inequality

^e^: Confidence Interval

Supplementary Table 11. The Slope Index of Inequality (SII) and Relative Index of Inequality (RII) of socioeconomic factors in the prevalence of difficulty in speaking after multiple imputation (n = 21,924)

|  | **Age- and sex-adjusted model^a^** | | | | **Fully-adjusted model^b^** | | | |
| --- | --- | --- | --- | --- | --- | --- | --- | --- |
|  | **SII^c^** |  | **RII^d^** |  | **SII** |  | **RII** |  |
|  | 95% CI^e^ | P value | 95% CI | P value | 95% CI | P value | 95% CI | P value |
| Educational attainment | 0.04 | <0.01 | 2.44 | <0.01 | 0.02 | <0.01 | 1.64 | <0.01 |
|  | (0.03; 0.05) |  | (1.86; 3.21) |  | (0.01; 0.03) |  | (1.25; 2.15) |  |
| Equivalent annual household income | 0.04 | <0.01 | 2.77 | <0.01 | 0.01 | >0.05 | 1.45 | >0.05 |
|  | (0.02; 0.05) |  | (2.01; 3.83) |  | (-0.003; 0.02) |  | (1.02; 2.06) |  |
| Wealth | 0.05 | <0.01 | 3.79 | <0.01 | 0.04 | <0.01 | 2.79 | <0.01 |
|  | (0.04; 0.06) |  | (2.82; 5.10) |  | (0.03; 0.06) |  | (1.95; 4.00) |  |
| Pension type | 0.02 | <0.01 | 1.66 | <0.01 | 0.004 | >0.05 | 1.09 | >0.05 |
|  | (0.01; 0.03) |  | (1.25; 2.20) |  | (-0.01; 0.02) |  | (0.83; 1.44) |  |

^a^: Adjusted for age and sex. Each socioeconomic factor was separately included

^b^: Adjusted for age, sex and other socioeconomic factors

^c^: Slope Index of Inequality

^d^: Relative Index of Inequality

^e^: Confidence Interval

Supplementary Table 12. The Slope Index of Inequality (SII) of socioeconomic factors in the prevalence of oral frailty and its components after multiple imputation (n = 21,924)

|  | **SII^a^** | | | | | |
| --- | --- | --- | --- | --- | --- | --- |
|  | **For oral frailty** | **For oral frailty components^c^** | | | | |
|  |  | **For**  **fewer teeth** | **For difficulty in chewing** | **For difficulty in swallowing** | **For**  **dry mouth** | **For difficulty in speaking** |
|  | 95% CI^b^ | 95% CI^b^ | 95% CI^b^ | 95% CI^b^ | 95% CI^b^ | 95% CI^b^ |
| Educational attainment | 0.18 | 0.27 | 0.17 | 0.02 | 0.01 | 0.04 |
|  | (0.16; 0.21) | (0.24; 0.29) | (0.14; 0.19) | (0.001; 0.05) | (-0.01; 0.03) | (0.03; 0.05) |
| Equivalent annual household income | 0.19 | 0.23 | 0.18 | 0.02 | 0.06 | 0.04 |
|  | (0.16; 0.21) | (0.20; 0.25) | (0.15; 0.20) | (0.002; 0.05) | (0.04; 0.08) | (0.02; 0.05) |
| Wealth | 0.25 | 0.34 | 0.22 | 0.02 | 0.07 | 0.05 |
|  | (0.22; 0.27) | (0.31; 0.36) | (0.20; 0.25) | (-0.0004; 0.04) | (0.05; 0.09) | (0.04; 0.06) |
| Pension  type | 0.12 | 0.15 | 0.12 | 0.02 | 0.01 | 0.02 |
|  | (0.09; 0.14) | (0.13; 0.18) | (0.09; 0.14) | (0.001; 0.05) | (-0.01; 0.04) | (0.01; 0.03) |

^a^: Slope Index of Inequality of socioeconomic factors in the prevalence of oral frailty and its components. All models were adjusted for age and sex, and each socioeconomic factor was separately included.

^i^: Confidence Interval

Supplementary Table 13. Changes in the Slope Index of Inequality (SII) when each component of oral frailty was separately added to the age- and sex-adjusted models after multiple imputation (n = 21,924)

| **Variables in the models** | **SII^a^** | **95% CI^b^** | **% reduction in SII compared to**  **age- and sex-adjusted model^c^** |
| --- | --- | --- | --- |
| Educational attainment |  |  |  |
| Age and sex | 0.18 | (0.16; 0.21)* |  |
| Age, sex, fewer teeth | 0.06 | (0.05; 0.08)* | 69.2% |
| Age, sex, difficulty in chewing | 0.07 | (0.05; 0.08)* | 63.5% |
| Age, sex, difficulty in swallowing | 0.17 | (0.15; 0.19)* | 6.4% |
| Age, sex, dry mouth | 0.18 | (0.15; 0.20)* | 3.4% |
| Age, sex, difficulty in speaking | 0.16 | (0.13; 0.18)* | 12.8% |
| Equivalent annual household income |  |  |  |
| Age and sex | 0.19 | (0.16; 0.21)* |  |
| Age, sex, fewer teeth | 0.08 | (0.06; 0.10)* | 56.5% |
| Age, sex, difficulty in chewing | 0.06 | (0.05; 0.08)* | 66.0% |
| Age, sex, difficulty in swallowing | 0.18 | (0.15; 0.20)* | 6.3% |
| Age, sex, dry mouth | 0.16 | (0.14; 0.18)* | 15.4% |
| Age, sex, difficulty in speaking | 0.16 | (0.14; 0.19)* | 12.0% |
| Wealth |  |  |  |
| Age and sex | 0.25 | (0.22; 0.27)* |  |
| Age, sex, fewer teeth | 0.09 | (0.07; 0.11)* | 63.5% |
| Age, sex, difficulty in chewing | 0.09 | (0.07; 0.11)* | 62.7% |
| Age, sex, difficulty in swallowing | 0.24 | (0.21; 0.26)* | 4.3% |
| Age, sex, dry mouth | 0.21 | (0.19; 0.23)* | 14.3% |
| Age, sex, difficulty in speaking | 0.21 | (0.19; 0.24)* | 13.4% |
| Pension type |  |  |  |
| Age and sex | 0.12 | (0.09; 0.14)* |  |
| Age, sex, fewer teeth | 0.05 | (0.02; 0.07)* | 60.8% |
| Age, sex, difficulty in chewing | 0.04 | (0.02; 0.06)* | 69.0% |
| Age, sex, difficulty in swallowing | 0.11 | (0.08; 0.13)* | 10.1% |
| Age, sex, dry mouth | 0.11 | (0.09; 0.13)* | 5.6% |
| Age, sex, difficulty in speaking | 0.11 | (0.08; 0.13)* | 9.4% |
| *: Statistically significant associations were identified at p <0.01 | | | |
| ^a^: Slope Index of Inequality | | | |
| ^b^: Confidence Interval | | | |
| ^c^: Percentage reduction in SII = ((SII_socioeconomic factor, controlling for age and sex_ - SII_socioeconomic factor_, _controlling for age, sex and one oral frailty component_)/ SII_socioeconomic factor, controlling for age and sex_)) x 100 | | | |

Supplementary Table 14. Demographic characteristics of participants, stratified by the prevalence of oral frailty by complete case analysis (n = 15,114)

| **Characteristics** | **Oral Frailty^a^** | | |
| --- | --- | --- | --- |
|  | **Absence** | **Presence** | **Overall** |
|  | **(row%^b^)** | **(row%^b^)** | **No. (col%^c^)** |
|  | **(n = 10,361)** | **(n = 4,753)** | **(n = 15,114)** |
| **Age** |  |  |  |
| 65-69 years | 77.0% | 23.0% | 3,826 (25.3%) |
| 70-74 years | 70.6% | 29.4% | 4,756 (31.5%) |
| 75-79 years | 65.1% | 34.9% | 3,412 (22.6%) |
| 80-84 years | 61.7% | 38.3% | 2,142 (14.2%) |
| ≥85 years | 53.0% | 47.0% | 978 (6.4%) |
| **Sex** |  |  |  |
| Male | 67.9% | 32.1% | 7,974 (52.8%) |
| Female | 69.3% | 30.7% | 7,140 (47.2%) |
| **Educational attainment** |  |  |  |
| ≥13 years | 74.1% | 25.9% | 5,698 (37.7%) |
| 10-12 years | 68.0% | 32.0% | 6,791 (44.9%) |
| ≤9 years | 58.1% | 41.9% | 2,625 (17.4%) |
| **Equivalent annual household income** |  |  |  |
| ≥4.0 million yen | 76.2% | 23.9% | 2,000 (13.2%) |
| 3-3.99 million yen | 73.9% | 26.1% | 2,492 (16.5%) |
| 2-2.99 million yen | 69.8% | 30.2% | 3,798 (25.1%) |
| 1-1.99 million yen | 65.6% | 34.4% | 5,314 (35.2%) |
| <1 million yen | 56.8% | 43.2% | 1,510 (10.0%) |
| **Wealth** |  |  |  |
| ≥50 million yen | 77.3% | 22.7% | 2,285 (15.1%) |
| 10-49.9 million yen | 71.8% | 28.2% | 6,665 (44.1%) |
| 5-9.99 million yen | 66.5% | 33.5% | 2,474 (16.4%) |
| 1-4.99 million yen | 61.1% | 38.9% | 2,322 (15.4%) |
| <1 million yen | 54.8% | 45.2% | 1,368 (9.0%) |
| **Pension type** |  |  |  |
| Rich^d^ | 73.3% | 26.7% | 3,356 (22.2%) |
| Moderate^e^ | 68.6% | 31.4% | 8,859 (58.6%) |
| Poor^f^ | 63.2% | 36.8% | 2,738 (18.1%) |
| None^g^ | 59.0% | 41.0% | 161 (1.1%) |
| ^a^: Oral frailty was defined by the presence of two or more of these components: fewer teeth, difficulty in chewing, difficulty in swallowing, dry mouth, and difficulty in speaking  ^b^: Row percentage  ^c^: Column percentage  ^d^: Enrolled in Corporate Pension Plan or Personal Pension Plan, either with or without National Pension Plan, Employees' Pension Plan or Mutual-Aid Pension Plan | | | |
| ^e^: Enrolled in Employees' Pension Plan or Mutual-Aid Pension Plan, either with or without National Pension Plan  ^f^: Enrolled in National Pension Plan  ^g^: No pension benefits | | | |

Supplementary Table 15. The Slope Index of Inequality (SII) and Relative Index of Inequality (RII) of socioeconomic factors in the prevalence of oral frailty by complete case analysis (n = 15,114)

|  | **Age- and sex-adjusted model^a^** | | | | **Fully-adjusted model^b^** | | | |
| --- | --- | --- | --- | --- | --- | --- | --- | --- |
|  | **SII^c^** |  | **RII^d^** |  | **SII** |  | **RII** |  |
|  | 95% CI^e^ | P value | 95% CI | P value | 95% CI | P value | 95% CI | P value |
| Educational attainment | 0.16 | <0.01 | 1.65 | <0.01 | 0.09 | <0.01 | 1.33 | <0.01 |
|  | (0.13; 0.19) |  | (1.51; 1.80) |  | (0.07; 0.12) |  | (1.21; 1.45) |  |
| Equivalent annual household income | 0.16 | <0.01 | 1.68 | <0.01 | 0.06 | <0.01 | 1.20 | <0.01 |
|  | (0.13; 0.19) |  | (1.54; 1.83) |  | (0.03; 0.09) |  | (1.09; 1.32) |  |
| Wealth | 0.23 | <0.01 | 2.02 | <0.01 | 0.17 | <0.01 | 1.67 | <0.01 |
|  | (0.20; 0.25) |  | (1.86; 2.19) |  | (0.14; 0.20) |  | (1.52; 1.84) |  |
| Pension type | 0.11 | <0.01 | 1.43 | <0.01 | 0.05 | <0.01 | 1.20 | <0.01 |
|  | (0.08; 0.14) |  | (1.30; 1.58) |  | (0.02; 0.08) |  | (1.09; 1.32) |  |

^a^: Adjusted for age and sex. Each socioeconomic factor was separately included

^b^: Adjusted for age, sex and other socioeconomic factors

^c^: Slope Index of Inequality

^d^: Relative Index of Inequality

^e^: Confidence Interval

Supplementary Table 16. Changes in the Slope Index of Inequality (SII) when each component of oral frailty was separately added to the age- and sex-adjusted models by complete case analysis (n = 15,114)

| **Variables in the models** | **SII^a^** | **95% CI^b^** | **% reduction in SII compared to**  **age- and sex-adjusted model^c^** |
| --- | --- | --- | --- |
| Educational attainment |  |  |  |
| Age and sex | 0.16 | (0.13; 0.19)* |  |
| Age, sex, fewer teeth | 0.05 | (0.03; 0.08)* | 67.5% |
| Age, sex, difficulty in chewing | 0.07 | (0.04; 0.09)* | 59.9% |
| Age, sex, difficulty in swallowing | 0.15 | (0.13; 0.18)* | 5.8% |
| Age, sex, dry mouth | 0.16 | (0.13; 0.18)* | 3.0% |
| Age, sex, difficulty in speaking | 0.14 | (0.11; 0.17)* | 14.0% |
| Equivalent annual household income |  |  |  |
| Age and sex | 0.16 | (0.14; 0.19)* |  |
| Age, sex, fewer teeth | 0.08 | (0.05; 0.10)* | 53.9% |
| Age, sex, difficulty in chewing | 0.05 | (0.03; 0.07)* | 66.5% |
| Age, sex, difficulty in swallowing | 0.15 | (0.13; 0.17)* | 7.3% |
| Age, sex, dry mouth | 0.13 | (0.11; 0.16)* | 17.9% |
| Age, sex, difficulty in speaking | 0.14 | (0.12; 0.17)* | 13.7% |
| Wealth |  |  |  |
| Age and sex | 0.23 | (0.20; 0.25)* |  |
| Age, sex, fewer teeth | 0.09 | (0.07; 0.11)* | 60.7% |
| Age, sex, difficulty in chewing | 0.09 | (0.07; 0.11)* | 61.3% |
| Age, sex, difficulty in swallowing | 0.22 | (0.19; 0.24)* | 5.5% |
| Age, sex, dry mouth | 0.19 | (0.17; 0.21)* | 16.3% |
| Age, sex, difficulty in speaking | 0.19 | (0.17; 0.22)* | 14.5% |
| Pension type |  |  |  |
| Age and sex | 0.11 | (0.08; 0.14)* |  |
| Age, sex, fewer teeth | 0.05 | (0.02; 0.07)* | 59.0% |
| Age, sex, difficulty in chewing | 0.03 | (0.01; 0.05)* | 73.8% |
| Age, sex, difficulty in swallowing | 0.10 | (0.07; 0.13)* | 9.9% |
| Age, sex, dry mouth | 0.10 | (0.08; 0.13)* | 5.8% |
| Age, sex, difficulty in speaking | 0.10 | (0.07; 0.13)* | 11.2% |
| *: Statistically significant associations were identified at p <0.01 | | | |
| ^a^: Slope Index of Inequality | | | |
| ^b^: Confidence Interval | | | |
| ^c^: Percentage reduction in SII = ((SII_socioeconomic factor, controlling for age and sex_ - SII_socioeconomic factor_, _controlling for age, sex and one oral frailty component_)/ SII_socioeconomic factor, controlling for age and sex_)) x 100 | | | |

Supplementary Figure Legend

Supplementary Figure 1. Flowchart of the study participants

Supplementary Figure 2. Theoretical framework using directed acyclic graph (DAG) for the socioeconomic inequalities in oral frailty and its components including confounders (age and sex)

Supplementary Figure 3. Contribution of inequalities in each oral frailty component to the overall inequality in oral frailty, by educational attainment (n = 21,924)

Supplementary Figure 4. Contribution of inequalities in each oral frailty component to the overall inequality in oral frailty, by equivalent annual household income (n = 21,924)

Supplementary Figure 5. Contribution of inequalities in each oral frailty component to the overall inequality in oral frailty, by wealth (n = 21,924)

Supplementary Figure 6. Contribution of inequalities in each oral frailty component to the overall inequality in oral frailty, by pension type (n = 21,924)

Supplementary Figure 7. The Slope Index of Inequality illustrating the estimated prevalence of oral frailty, fewer teeth, difficulty in chewing, difficulty in swallowing, dry mouth, and difficulty in speaking by educational attainment, equivalent annual household income, wealth, and pension type by complete case analysis (n = 15,114)

Supplementary Figure 1. Flowchart of the study participants

Target population of the JAGES 2022 survey

(n = 309,833)

Non-respondents

(n = 103,629)

Total respondents

(n = 206,204;

response rate = 66.6%)

Invalid responses or lack of informed consent for research use

(n = 13,159)

Valid responses

(n = 193,045)

Respondents who answered any of the other seven versions not including oral health behaviors questions

(n = 168,745)

or lacked version information

(n = 208)

Valid responses from Version D – related to oral health behaviors questions

(n = 24,092)

Activities of daily living were not independent or lacked of this information

(n = 2,168)

Analyzed population

(n = 21,924)

Supplementary Figure 2. Theoretical framework using directed acyclic graph (DAG) for the socioeconomic inequalities in oral frailty and its components including confounders (age and sex)

**Confounders**

Age, sex

**Oral frailty and its components** (fewer teeth, difficulty in chewing, difficulty in swallowing, dry mouth, difficulty in speaking)

**Socioeconomic factors**

Educational attainment

Equivalent annual household income

Wealth

Pension types

Supplementary Figure 3. Contribution of inequalities in each oral frailty component to the overall inequality in oral frailty, by educational attainment (n = 21,924)

|  | % reduction in SII |
| --- | --- |
| Fewer teeth | 69.2 |
| Difficulty in chewing | 63.5 |
| Difficulty in swallowing | 6.4 |
| Dry mouth | 3.4 |
| Difficulty in speaking | 12.8 |

Supplementary Figure 4. Contribution of inequalities in each oral frailty component to the overall inequality in oral frailty, by equivalent annual household income (n = 21,924)

|  | % reduction in SII |
| --- | --- |
| Fewer teeth | 56.5 |
| Difficulty in chewing | 66 |
| Difficulty in swallowing | 6.3 |
| Dry mouth | 15.4 |
| Difficulty in speaking | 12 |

Supplementary Figure 5. Contribution of inequalities in each oral frailty component to the overall inequality in oral frailty, by wealth (n = 21,924)

|  | % reduction in SII |
| --- | --- |
| Fewer teeth | 63.5 |
| Difficulty in chewing | 62.7 |
| Difficulty in swallowing | 4.3 |
| Dry mouth | 14.3 |
| Difficulty in speaking | 13.4 |

Supplementary Figure 6. Contribution of inequalities in each oral frailty component to the overall inequality in oral frailty, by pension type (n = 21,924)

|  | % reduction in SII |
| --- | --- |
| Fewer teeth | 60.8 |
| Difficulty in chewing | 69 |
| Difficulty in swallowing | 10.1 |
| Dry mouth | 5.6 |
| Difficulty in speaking | 9.4 |

Supplementary Figure 7. The Slope Index of Inequality illustrating the estimated prevalence of oral frailty, fewer teeth, difficulty in chewing, difficulty in swallowing, dry mouth, and difficulty in speaking by educational attainment, equivalent annual household income, wealth, and pension type by complete case analysis (n = 15,114)
